# Reliability and Maintenance Analysis of Unmanned Aerial Vehicles †

**DOI:** 10.3390/s18093171

**Published:** 2018-09-19

**Authors:** Enrico Petritoli, Fabio Leccese, Lorenzo Ciani

**Affiliations:** 1 Science Department, Università degli Studi “Roma Tre”, Via della Vasca Navale n. 84, 00146 Rome, Italy; e_petritoli@libero.it; 2 Department of Information Engineering, University of Florence, Via S. Marta n. 3, 50139 Florence, Italy; lorenzo.ciani@unifi.it

**Keywords:** UAV, unmanned, aerial, vehicle, RAMS, reliability, maintainability, preventive, corrective, maintenance, hard failure, soft failure, uncertainty, confidence interval

## Abstract

This paper focuses on the development of a new logistic approach based on reliability and maintenance assessment, with the final aim of establishing a more efficient interval for the maintenance activities for Unmanned Aerial Vehicles (UAV). In the first part, we develop an architectural philosophy to obtain a more detailed reliability evaluation; then, we study the intrinsic reliability at the design stage in order to avoid severe critical issues in the UAV. In the second part, we compare different maintenance philosophies for UAVs and develop the concepts of preventive and corrective maintenance that consider the system subjected (until real “hard failure”) to partial performance degradation (“soft failure”). Finally, by evaluation of the uncertainty through the confidence interval, we determine the new soft failure limits, taking into account the general knowledge of the systems and subsystems in order to guarantee the proper preventive maintenance interval.

## 1. Introduction

The problem of reliability of UAVs, like problems of maintenance and safety, have become extremely important in recent years: engines became more robust, avionics was improved, etc. Despite this, the approach regarding the reliability of drones is still too fatalistic.

Obviously, by means of reliability analyses which are available nowadays, we consider that the absence of a driver or person on board does not allow us to design and realize the UAV with less stringent standards with respect to those used for airplanes. The commercial aviation failure rate is about 1/10^5^ flight hours, while for drones, it has been verified at about 1/10^3^ flight hours, so a higher magnitude of two orders. From a different point of view, sophisticated UAV systems have an overall failure rate of 25%. The aim of the paper, which is an extended version of [1], is to provide new ideas to increase the reliability of a drone optimizing maintenance activities. For this, we start from the philosophy of apportioning the percentages of reliability assigned (on average) to each system (and subsystem), trying to optimize them according to safety requirements. On the other hand, it is necessary to optimize the time intervals (and consequently the costs) of maintenance, taking into account that all critical systems must absolutely support preventive maintenance criteria: in these cases, we are helped by the concepts of *soft* and *hard* failure [2,3].

### 1.1. Definitions

Here, we will introduce a series of definitions which will be used throughout the paper.

#### 1.1.1. Reliability

Reliability is a dynamic concept which is applicable to many fields, i.e., is not only strictly technical. For it, a possible definition could be expressed in terms of probability, and in particular, as “the probability that a system, subsystem or part is able to perform its specific function in a pre-established time and under pre-established conditions”. One of the most important reliability metrics is represented by the Mean Time Between Failures (MTBF), expressed in hours of activity; the higher the value, the more reliable the equipment. For a part or a single subsystem, the MTBF is often expressed as the reciprocal of reliability. For instance, the MTBF gives information about the level of unreliability, and it typically shows the number of failures of a piece of equipment over an established time, i.e., 10,000 h [4].

The Failure In Time (FIT) rate of a device represents the number of expected failures in one billion (10^9^) device-hours of operation. This parameter is widely diffused in the semiconductor industry [5,6,7] and international standards.

#### 1.1.2. Availability

This parameter is extremely important for ‘ready to operate’ system. It measures the number of times for which the system under study is available or ready with respect to the number of times in which the system is required. Typically, this parameter is presented in form of a percentage, where 100% is the theoretical goal [8,9,10,11].

#### 1.1.3. The Environment

According to “MIL-HDBK-217F2”, [12] (see Table 1), the following operative environment is considered for the reliability prediction:

## 2. RAMS Assessment

The *Reliability, Availability, Maintainability, and Safety (RAMS)* assessment is an important study in the development of UAVs. This kind of analysis is mandatory if you want to increase the reliability of a drone, its availability, and to reduce repair and maintenance costs [13]. Once an architecture has been chosen, the RAMS assessment is very useful to identify all the critical elements that could increase the failure rate [14]. It also allows us to characterize all the most stressed (or undersized) areas of the project. Furthermore, the reliability prediction, for example, makes it possible to decide whether to duplicate a safety critical system or to put it in *derated* conditions, with great savings in terms of weight and power consumption [15,16,17,18,19]. A comparison between the well-known but always efficient technique of redundancy and the improvement of reliability must consider important remarks such as norms, costs, limitations of spaces, and so on. The reliability analysis helps us to assess the value of failures [20]. For instance, if some failures of a specific component happen in a wider system, the failure rate, preventively predicted, is useful to establish if the number of failures which is adequate to the overall number of components present in the system. Alternatively, it can individuate a particularly problematic section [21,22,23,24]. Finally, this kind of evaluation can be used to assess the probabilities of findable damage events in a FMECA (Failure Modes, Effects and Criticality Analysis).

## 3. How Reliable Does a Drone Have to Be?

During a typical mission, some failures are more critical than others: the loss of longitudinal stability, the loss of payload data, or the turning off of the position lights are not of the same criticality level. Therefore, it is necessary first to establish various levels of increasing gravity associated with the fault [25]. Moreover, according to the specific kind of mission and the specific kind of UAV, it is necessary to subdivide failures into subcategories [26]. Then, for each scenario, suitability and preventively forecasted, it is necessary to define a minimum acceptable level of reliability. Finally, even for the aircraft, it is necessary to define the criteria for the level of reliability, in terms of a level which is strictly linked to the type of failure [27,28,29,30,31].

- *Catastrophic failures*: for these kind of failures, a crash of the drone is certain while injuries or even the death of persons on the ground is possible.
- *Severe failures*: heavy damages are expected and the probability of repairing the drone is low.
- *Moderate failures*: cause a moderate degradation of the drone’s functions, which could lead to aborting the mission; however, they are not cause of severe damage.
- *Soft failures*: cause light degradation of the drone’s functions, but do not lead to the cancellation of the mission.

The *intrinsic reliability* of an apparatus (in our case, a drone) is the reliability studied *a priori* [32]; however, unfortunately, a reliability study is often realized *after* the design phase. This approach can lead to many problems during the management of the project, because a reliability study highlights a series of sensible points, and produces a series of recommendations that are usually forwarded to the designers. These should allow them to carry all the necessary modifications to the project in order to improve it [33,34,35,36,37,38]. The perspective is completely different in the case of intrinsic reliability: in fact, the knowledge of the failure distribution of a system gives rise to the possibility, directly during the design stage, of taking specific action to reduce criticality and upgrade the critical parts or subsystems in advance, thereby increasing the overall level of reliability [39]. This, surely, increases the level of responsibility of the designers, but at the same time, decreases the risk of criticalities (also called Single Point Failures or briefly SPF) that might happen in future studies [40,41,42,43].

This way of seeing the design phase, i.e., in which a reliability study is effectively used to help the project, means that the figure of the Quality Assurance Responsible is frequently present from the beginning. Reliability analyses primarily aim to find the minimum limits for the requirements that allow UAVs to have at least a magnitude of better reliable [44].

**Figure 1:**
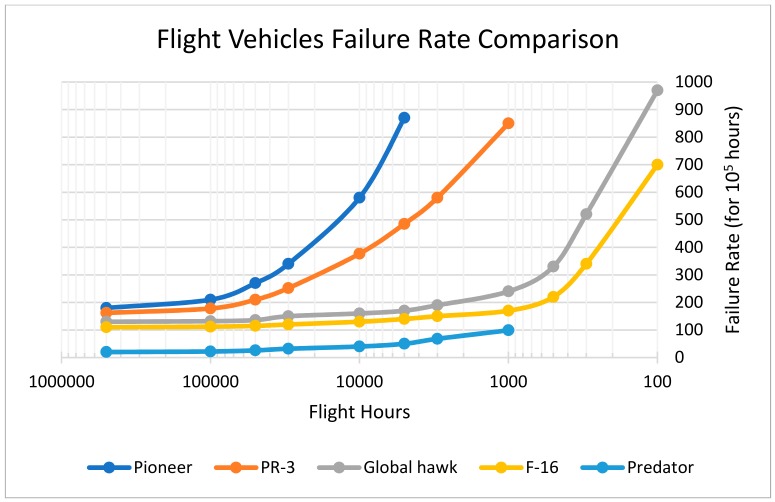
Failure Rate vs. Flight hours for the F-16 and some common drones (figure extrapolated on the data of: Barnard Microsystems Inc. et al. [5]).

Another benefit of these analyses is that they help us to understand which components or parts of a specific subsystem are the most unreliable, and which are the most critical to the system [45,46,47,48,49,50]. In Figure 1 a failure rate comparison between five different aircrafts is shown: Northrop Grumman RQ-4 Global Hawk in gray, PR-3 in orange, General Atomics RQ-1 Predator in blue, AAI RQ-2 Pioneer (for the drone category) in light blue and General Dynamics F-16 jet fighter in yellow.

## 4. Reliability Assessment Hierarchy

**Figure 2:**
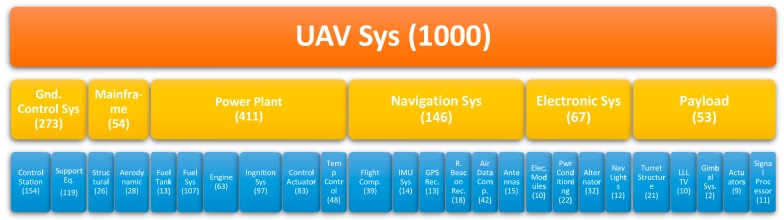
The Hierarchy of the Reliability Assessment (every 10^3^ system failures) for UAVs. The structure is subdivided into main systems (yellow), subsystems (blue), all together representing the UAV system (orange); (figure extrapolated on the data of: Barnard Microsystems Inc. et al. [5]).

Considering all the possible main systems and subsystems that form an UAV, Figure 2 depicts the UAV hierarchy of the reliability assessment, showing their failure distribution every 10^3^ faults [51,52].

The following subparagraphs show how the failures are categorized, taking into account the function of each subsystem.

### 4.1. Ground Control System (GCS)

The Ground Control System, also called GCS, is the part with the highest maintainability of the whole UAV system; this is because it is easily accessible at any time during the mission. It is mainly composed of COTS (Commercial Off-The-Shelf) components with large inventories. This does not mean that it is less safe; the fact that it is ground-based allows the introduction of a good percentage of redundant systems with hot and cold stand-by configurations, reducing the off-line time nearly to zero [53].

### 4.2. Mainframe

The UAV mainframe is by far the strongest part of the whole system; this is designed with great attention by means of CAD systems, which allow the developers to study and evaluate the loads of the structure *a priori*. In general, the mainframe is appropriately oversized; in fact, even if this leads to extra weight, it is undoubtedly a small price to pay for a safer structural system. Generally, the most common failures occur due to fatigue cycles, soldering brazing, or untreated rivets [54].

### 4.3. Power Plant

The power plant itself is a rather reliable mechanical system, even if the subsystems could show some breakdowns. Especially in long-term missions, insufficient fuel vaporization or poor cooling can lead to overheating of the engine, or fatigue that could eventually lead to failure [55,56].

### 4.4. Navigation System

This system is the most important part of the vehicle; it is characterized by a high failure rate compared to other systems. Nevertheless, it has the highest number of hot redundant subparts/subsystems. Therefore, due to the high level of electronic miniaturization, it is possible to replicate a large number of its subsystems [57] making it intrinsically reliable. The Hot (standby) redundancy is a method in which one system runs simultaneously with an identical primary system. Upon failure of the primary system, the hot standby system immediately takes over, replacing the primary system.

Moreover, due to the strong integration derived from the experience of the development of automotive applications, the aerospace world enjoys highly-reliable electronic components for navigation (as Inertial Navigation System—INS and Global Positioning System—GPS receivers). A second benefit comes from their high computing capacity that allows a parallel computing architecture, greatly increasing the overall reliability [58].

### 4.5. Electronic System

In the previous discussion, we have deliberately separated the electronic system from the navigation system. Even from a purely mechanical point of view, it may not be so evident how they are separated from a functional and philosophical point of view. In fact, in order to prevent possible interference, the electronic system separates all the electronic circuits which are not closely related to navigation, such as the power supply and conditioning, to manage the telecommunication system to the outside (satellite communications, ground-vehicle data links, etc.). Even in this case, the hypothesis of redundancy has to be avoided because, for example, the weight of the harness would be excessively high for such a small vehicle [59].

### 4.6. Payload

The payload is not contained in its own conditioned bay inside the fuselage, but is placed outside in a mobile turret inserted directly in the aerodynamic flow. The turret itself contains several electro-optical sensors like a thermal imaging camera, a Low Light Level Television, a laser tracer etc. From a mechanical point of view, the turret is gimballed, allows ± 90° elevation, and 360° of continuous azimuth rotation; the system also contains the ancillary electronics of the sensors and the movement actuators.

The turret is thermostatically controlled to ensure optimal operation of the electronics and to prevent freezing of the kinematic devices in high-altitude flight conditions. For these applications, the electronics will be chosen with consideration of their intrinsic reliability and highest temperature operative range. The geometry of the cases of the components must be chosen carefully, as the aircraft is subjected to frequent and abrupt changes in altitude, and therefore, pressure, that could stress some components. It’s important to note that to put all the equipment into a sealed and pressurized container would be too burdensome from the point of view of weight.

## 5. Multiplexed Systems

Redundancy of the most critical systems does not always lead to an increase in the reliability of the whole system. In fact, the redundancy of a quite unreliable system means increases in the total failure rate. In other words, the overall safety of the system will be increased, but certainly not the reliability. A higher failure rate brings an increase in expenses for spare parts and in person-hours, increasing operating costs [11].

On the other hand, the duplication of the most critical or vital systems is not the ideal solution, as it increases the cost and the complications of the system, so it is necessary to find another way to improve reliability [60].

A classic case is that related to propulsion systems: many UAVs have only one engine, but this is a highly reliable system, even if its loss compromises the entire mission. The installation of two engines would seem to be an ideal solution because the loss of one of these would not compromise the final mission. However, the duplication of a motor means the duplication of all ancillary systems. This in turn leads to a decline in the system’s overall reliability [61].

The ideal solution is based on two keywords: *oversize* and *derating*. We will therefore choose an engine with characteristics that exceed the UAV requirements, and will work under ordinary operating conditions, i.e., derated, or in a very “relaxed” way. In this case, we will see that, while remaining a single point failure according to FMECA analysis, the engine has a considerably higher reliability, as it will work at less than 50% of its capability; this condition reduces the rate of failure occurrence.

## 6. The UAV as a Complex Maintenance System

Now we consider an UAV as a complex aerial system composed by *m* subsystems (or subparts) defined as J = {1, 2, …, *m*}, and consisting of *l_j_* components. At the component level, we can continuously control and check the degradation of a defined collection of physical parameters. The physical conditions degrade monotonically during use, and are restored by maintenance actions. For each component or subpart *i* ∈ *I*, *X_i_*(*t*) indicates the degradation trajectory in a fixed time interval *t* ∈ [0, ∞). Soft failure can be defined as the ability of a component, part, subsystem or system to continue its work even if with degraded performance, i.e., up to the point when its reduced performance exceeds a specific fixed threshold *H_i_* (with *X_i_*(*t*) > *H_i_*), called *soft failure* one. Typically, components subjected to thermal stress or mechanical degradation are hit by soft failures.

**Figure 3:**
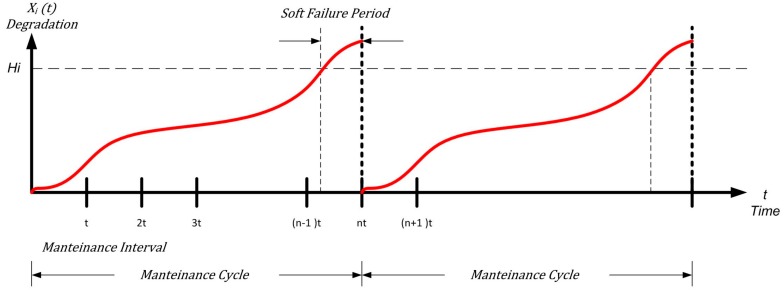
Degradation threshold of a system with a cycle of corrective maintenance only.

When *X_i_*(*t*) exceeds *H_i_*, a soft failure happens between two maintenance points (*n* − 1)*τ* and *nτ*. This implies an action of corrective maintenance (CM), which has a specific cost (*c_i_^CM^*) on the critical component. This action is executed in a fixed time called maintenance point *nτ*, as shown in Figure 3.

The period between the occurrence of the soft failure point and the maintenance point *nτ* is defined as “soft failure period”. This period defines loss of quality in production or poorer performance with a cost rate indicated with *c_i_^P^* [61].

### 6.1. Degradation Model for an UAV

In this section, starting from the UAV degradation model, we will arrive at defining limits and probabilistic criteria to determine the optimal maintenance interval that does not exceed the corrective maintenance threshold: maintenance that yields effect when the gradual damage is intolerable by the agreed-upon operational standards.

The random coefficient model is used to evaluate the level of degradation for the *i*th component for a time $\hat{t}$∈[0, ∞) in a cycle of single maintenance *Φ_i_* = {*ϕ_i,_*_1_, …, *ϕ_i,Q_*}, *Q* ∈ ℕ*,* then a set of random parameters *Θ_i_* = {*θ_i,_*_1_, …, *θi_,V_* }, *V* ∈ ℕ following a normal (Laplace–Gauss) distribution [62].

The probability that the degradation at time $T_{\chi }$ reaches the threshold χ before time $\hat{t}$ is:

(1) $$\;P_{r}\left\{T_{\chi }<\;\hat{t}\right\}=\;P_{r}\left\{X\left(\hat{t}\;;\Phi_{i},\Theta_{i}\;\right)>\chi \right\},\;\;\forall i\in I\;$$

The calculation of this probability is necessary because, in the next discussion, we will introduce a certain degradation profile and calculate the probability that this has to overcome the various critical failure thresholds.

We consider a complex system with the following degradation path (this type of degradation has been chosen because it is typical of this kind of complex systems):

(2) $$\;X_{i}\;\left(t,\;\Phi_{i},\Theta_{i}\right)=\phi_{i,1}+\theta_{i,1}\cdot {\hat{t}}^{\phi_{i,2}}\;$$

where $\Phi_{i}=\left\{\phi_{i,1},\;\phi_{i,2}\right\}$ and $\;\Theta_{i}=\left\{\theta_{i,1}\right\}$ then:

(3) $$\;P_{r}\left\{T_{\chi }<\hat{t}\right\}=\;P_{r}\left\{\phi_{i,1}+\theta_{i,1}\cdot {\hat{t}}^{\phi_{i,2}}>\chi \right\}\;$$

(4) $$\;P_{r}\left\{T_{\chi }<\hat{t}\right\}=\;P_{r}\left\{\theta_{i,1}>\frac{\chi \;-\;\phi_{i,1}\;}{{\hat{t}}^{\phi_{i,2}}}\right\}\;$$

For a random variable, $\theta_{i,1}\geq 0$, we evaluate the cumulative density function $F_{\theta_{i,1}}\;$:

(5) $$\;P_{r}\left\{T_{\chi }<\hat{t}\right\}=\;1-\;F_{\theta_{i,1}}\left(\frac{\chi \;-\;\phi_{i,1}\;}{{\hat{t}}^{\phi_{i,2}}}\right)\;$$

Now we evaluate the probability in which, between the two time points (*n* − 1)*τ* and *nτ,* the control limit *C_i_* is reached:

(6) $$\;P_{r}\left\{X_{i}\left(\left(n-1\right)\tau ;\;\Phi_{i},\Theta_{i}\right)\right\}\leq C_{i}<X_{i}\left(n\tau ;\Phi_{i},\Theta_{i}\;\right),\;\;\forall n\in \mathbb{N}\;$$

that is equal to:

(7) $$\;P_{r}\left\{\left(n-1\right)\tau \right\}\leq T_{C_{i}}<n\tau ,\;\;\forall n\in \mathbb{N}\;$$

The soft failure threshold *H_i_* is reached before time point *nτ* only if it has satisfied the following condition:

(8) $$\;P_{r}\left\{X_{i}\left(n\tau ;\Phi_{i},\Theta_{i}\;\right)>H_{i}\right\}=P_{r}\left\{T_{H_{i}}<n\tau \right\},\;\;\;i\in I\;$$

Moreover, assuming the degradation path as monotonic (typical of this kind complex systems), we have: *C_i_ < H_i_* with and $T_{C_{i}}\leq \;T_{H}$.

### 6.2. Uncertainty of Degradation in Corrective Maintenance

**Figure 4:**
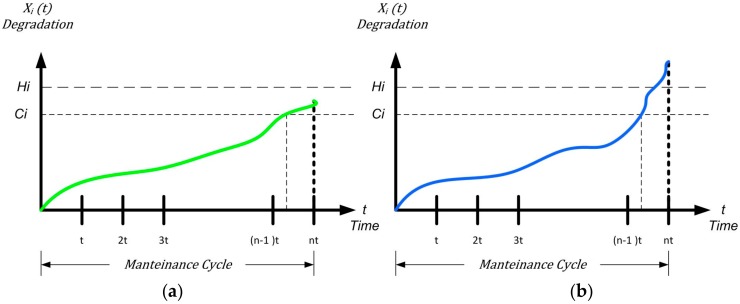
(**a**) Maintenance limit of preventive maintenance; (**b**) maintenance limit corrective maintenance.

If $\left(n-1\right)\cdot \tau \;\leq C_{i}<n\tau$ we have two occurrences for the maintenance decision at time *nτ: preventive* maintenance (PM) (see Figure 4a) and *corrective* maintenance (CM) (see Figure 4b) according to:

(9) $$\;\{\begin{array}{l} Preventive\;\;if:\;C_{i}\;\;\leq X_{i}\left(n\tau \right)<H_{i}\\ Corrective\;\;if:\;\;X_{i}\left(n\tau \right)\geq H_{i} \end{array}\;$$

The probability that a preventive maintenance happens at the specific time *nτ* after the degradation level of the *i*th component has reached the control limit $\left(n-1\right)\cdot \tau \;\leq C_{i}<n\tau$ is [63]:

(10) $$\;P_{r}\left\{PM\;\;at\;\;n\tau \right\}=\;P_{r}\left\{T_{H_{i}}>n\tau ,\;\left(n-1\right)\tau \leq T_{C_{i}}<n\tau \right\}\;$$

**Figure 5:**
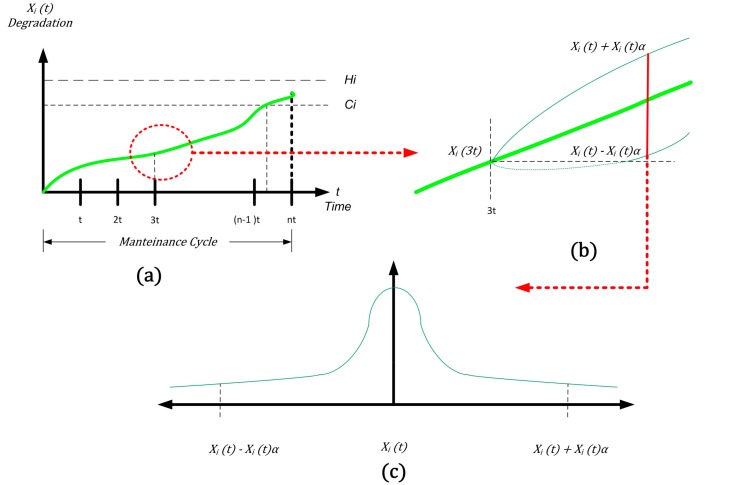
Uncertainty evaluation of corrective maintenance: the inspection point at 3t in detailed and expanded as a confidence interval.

Now we consider the monotonic expression in the preventive maintenance: consider, for example, what is happening around the monotone function immediately after the *3t* maintenance interval (see Figure 5a).

After the maintenance interval, since one of the returns from the field of intervention is the degradation state of the systems and subsystems, we know exactly what the degradation status of the systems and subsystems is. In other words, we can quantify as *X_i_*(*3t*) as the status of the probability at the moment we are studying.

Immediately after the “3t moment”, we have a view of the *drift in time* of the value: a band of uncertainty affects the probabilistic function (supposedly monotonous). The uncertainty is due to the capability of controlling the state of degradation of systems (and subsystems) limited by our confidence interval (see Figure 5a–c) in terms of knowledge of the complete system.

Now we consider *X_1_*, *…*, *X_n_* as samples of the subsystems degradation status of normal density after the revision *X_i_*(*3t*) with unknown mean *m* and variance σ^2^ (known) and sample average $\bar{X}$. We have:

(11) $$\;\overset{Xi\left(t\right)+Xi\left(t\right)\;\alpha }{\underset{Xi\left(t\right)-Xi\left(t\right)\;\alpha }{\int }}\frac{1}{\sqrt{2\pi }}e^{\frac{Xi\left(t\right){\;}^{2}}{2}}\;dX\;=Xi\left(t\right)\;\left(1-\alpha \right)\;$$

Therefore, we have:

(12) $$\;Xi\left(t\right)\left(1-\alpha \right)=\;P_{r}\left\{\left|\frac{\sqrt{n}}{\sigma }\cdot \left(\bar{X}-m\right)\right|\leq \;Xi\left(t\right)\;\alpha \right\}\;$$

**Figure 6:**
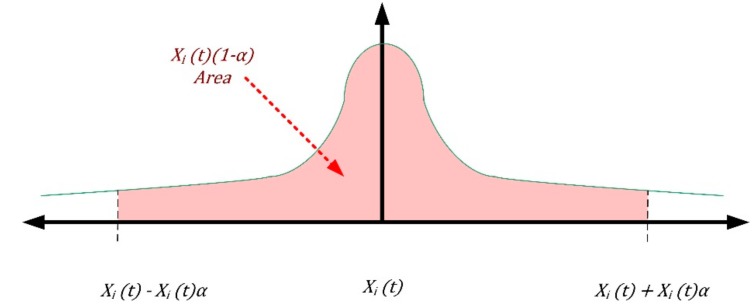
Confidence interval area considered for uncertainty.

Explaining the second member (see Figure 6):

(13) $$\;Xi\left(t\right)\left(1-\alpha \right)=\;P_{r}\left\{\bar{X}-\;\frac{\sigma }{\sqrt{n}}\cdot Xi\left(t\right)\alpha \leq m\;\leq \bar{X}+\;\frac{\sigma }{\sqrt{n}}\cdot Xi\left(t\right)\;\alpha \right\}\;$$

We have for the confidence interval of level Xi(t)(1−α)= for *m*:

(14) $$\;\left[\bar{X}-\;\frac{\sigma }{\sqrt{n}}\cdot Xi\left(t\right)\alpha ,\bar{X}+\;\frac{\sigma }{\sqrt{n}}\cdot Xi\left(t\right)\;\alpha \;\right]\;$$

It is necessary to treat the limitation of this method: the term Xi(t)−Xi(t) α can never be lower than *X_i_*(*3t*). This is because the system, despite monotonic evolution in a more optimistic than linearized way (Figure 5b—green line), cannot, for logical reasons of entropy, improve over time, or have a *negative* degradation. This condition is only theoretically possible, and is due to the uncertainty of the state of knowledge of the system. Therefore, to restore the physical consistency of the uncertainty evaluation, it is necessary to add the condition:

(15) $$\;\{\begin{array}{c} \left[\bar{X}-\;\frac{\sigma }{\sqrt{n}}\cdot Xi\left(t\right)\alpha ,\bar{X}+\;\frac{\sigma }{\sqrt{n}}\cdot Xi\left(t\right)\;\alpha \;\right]\\ Xi\left(3t\right)\leq Xi\left(t\right)-Xi\left(t\right)\;\alpha \end{array}\;$$

Our prediction of the state of health of our system cannot disregard the knowledge of the state of the subsystems: their number and state influence the possible uncertainty of the value *X_i_*(*t*).

### 6.3. The Thresholds of Preventive Maintenance

Considering the above calculations, when we want to evaluate the occurrence of the preventive maintenance intervals, we need to consider the confidence interval in terms of knowledge of the subsystems.

Reconsidering the confidence interval and the *n* cycle, the second part of the expression (9) becomes:

(16) $$\;Xi\left(nt\right)+Xi\left(nt\right)\;\alpha <H_{i}\;$$

Obviously, the real problem happens when the threshold $H_{i}$ is exceeded:

(17) $$\;Xi\left(nt\right)+Xi\left(nt\right)\;\alpha <H_{i}\;$$

so:

(18) $$\;Xi\left(nt\right)\;\left(1+\alpha \right)<H_{i}\;$$

And the *new sof failure* limit is:

(19) $$\;Xi\left(nt\right)\;<\frac{H_{i}}{1+\alpha }\;$$

Therefore, it is necessary to keep the confidence interval as narrow as possible.

Knowledge of the subsystems is therefore essential for evaluation: we risk calculating the total reliability of the system without evaluating the total accuracy that, in the worst case, would lead to a wrong assessment of preventive maintenance or an incorrect evaluation of preventive maintenance. Obviously, this is an undesirable situation.

The condition for the threshold $\;C_{i}$ is:

(20a) $$\;\;C_{i}\;\leq \;Xi\left(nt\right)-Xi\left(nt\right)\;\alpha \;$$

so:

(20b) $$\;\;C_{i}\;\leq \;Xi\left(nt\right)\;\left(1-\alpha \right)\;$$

and:

(21) $$\;\;\frac{\;C_{i}\;}{1-\alpha }\;\leq \;Xi\left(nt\right)\;$$

Now, the first member of the (9) becomes:

(22) $$\;\frac{\;C_{i}\;}{1-\alpha }\;\leq \;Xi\left(nt\right)\;<\frac{H_{i}}{1+\alpha }\;$$

Now we can objectively quantify the level of accuracy needed to define the preventive maintenance intervals.

**Figure 7:**
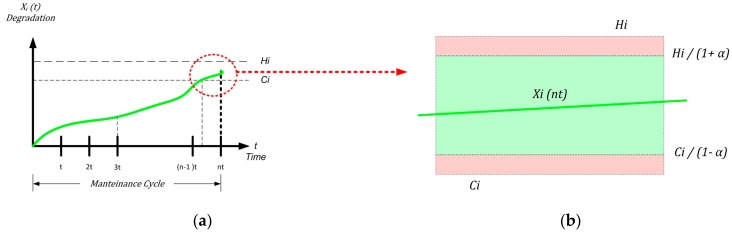
Uncertainty evaluation of corrective maintenance: original interval area (**red**) and the evaluation of the confidence interval (**green**).

It is still useful to specify the function from a graphical point of view. Now consider the upper zone of Figure 4a in detail (see Figure 7):

From Figure 7a, the correlation between confidence and useful interval for preventive maintenance is evident (in Figure 7b the detail is enlarged and the confidence intervals evidenced): the lower the confidence, the higher the probability that corrective maintenance is necessary.

### 6.4. The Failure Rate Paradox

Now let’s examine the real case of two completely different drones: a commercial drone and a surveillance drone. Both have on board, as payload, a system of stabilized cameras: in our reliability study, we will examine and compare only the systems and subsystems which they have in common.

Let us now compare the reliability of the average commercial drone: a reliability profile has been created as a weighted average of the data present in our database, created through previous research. This is compared to an “average” military drone created according to official sources [64]. Furthermore, the reliability of all subsystems is compared; it is immediately evident that the distribution is different.

According to Table 2, it is absolutely evident that the military drone, due to its complexity, has a reliability that is considerably inferior to that of a commercial drone which is certainly not built with stringent parameters and requirements.

As is known, the commercial drone is composed entirely of COTS parts. Although there are not, for example, “MIL” reliability-level electronic components, the reliability of commercial electronic components is now extremely high, even those with plastic casing. This is also a consequence of the continuous research in the automotive field, where the operating temperature range is quite high. All of these aspects, combined with low construction complexity, lead to a high reliability level.

The military drone, on the other hand, enjoys a high degree of redundancy and a knowledge of high quality components, but being an extremely sophisticated and complex product, it is heavily penalized from the point of view of the reliability figure.

It must stated, however, that the capabilities of the latter compared to the commercial drone are noteworthy: greater range, greater autonomy, higher payload, and resistance to soft failure. These are all characteristics that are transparent to the calculation of reliability, and that then, eventually, lead to the paradox.

**Figure 8:**
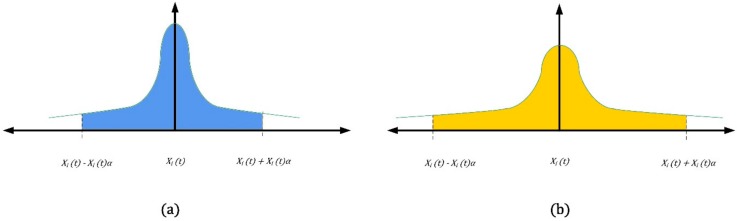
Confidence interval area considered for uncertainty: commercial drone (**blue**) and military (**yellow**).

In light of these considerations, we review, in Figure 8 (Figure 8a refers to “drone a” and Figure 8b refers to “drone b”), the previous confidence interval area considered for uncertainty in Figure 6:

Due to the good knowledge of the systems and subsystems of the military drone (hereafter referred to as “drone b”), we can have the basis for a much wider Gaussian, and conversely, the value of α.

**Figure 9:**
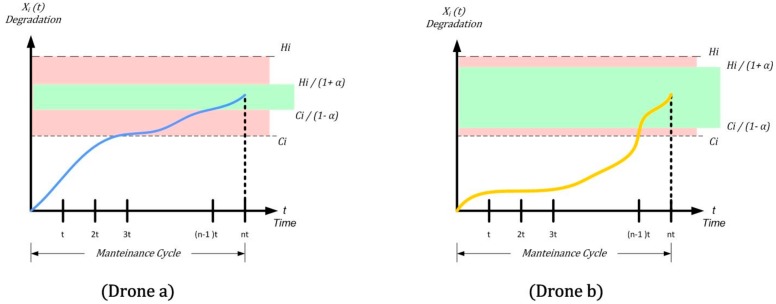
Uncertainty evaluation of corrective maintenance: original interval area (**red**) and the evaluation of the confidence interval (**green**).

Reviewing the interval in Figure 7, for the two different drones we have the following uncertainties (see Figure 9):

Considering “Drone a”, although its reliability is considerably higher, the knowledge of the components is lower. All this is reflected, from the analytical point of view, in the “shrinking” of the green band (see Figure 9), or the limits of preventive and corrective maintenance which are very close to each other. From the real point of view, this means that if we do not want to overcome the new limit $H_{i}/\left(1+\alpha \right)$, it is necessary to slightly reduce interval t, with a consequent increase of maintenance costs and a decrease in the general figure of availability.

The “Drone b”, (military) has, despite a bit of “shearing” by the α factor dropping a good margin, an increase in the frequency of the maintenance cycle, which will always remain modest.

## 7. Conclusions

In this paper, the uncertainty in the choice of the preventive maintenance intervals with respect to the soft failure threshold have been investigated, taking into account the reliability and safety requirements for Unmanned Aerial Vehicles (UAV).

First, we examined the state of the art of the philosophy of the UAVs and the roles and capabilities of operators. However, the increase of their use is strongly accompanied by higher failure rates compared to conventional, manned airplanes.

Then, we correlated the reliability of the drones with the maintenance intervals: a higher failure rate leads to very expensive repairs. In order to improve safety, the duplication of the troublesome elements is not the only solution. Therefore, it is necessary to obtain the required reliability level by also using high-quality, derated components, combined with a very detailed selection of a redundant subsystem during the design phase.

The innovation of our paper passes first through the review of the optimization of the probabilistic functions (under the conditions of a real case). Then, we find the optimal point of maintenance. It will be necessary to take into account a very large number of variables for all systems and subsystems in order to minimize uncertainty.

Finally, by evaluating uncertainty through the confidence interval, it is possible to accurately determine the maintenance intervals in order to not exceed the *new soft failure* limit, that takes into account the general knowledge of the systems and subsystems, and to remain always within the preventive maintenance limit time (and budget).

## Figures and Tables

**Table 1:** Environment definition.

Class ^1^	Definition ^1^
**A_UF_** Airborne, Uninhabited, Fighter	Environmentally uncontrolled areas, which cannot be inhabited by an aircrew during flight. Environmental extremes of pressure, temperature and shock may be severe.

^1^ Source: MIL-HDBK-217F2.

**Table 2:** Comparison between the reliability of a commercial and a military drone.

**Commercial Drone (a)**			
**System Description**	**λ_P_ System FIT (F/10^6^ hrs)**	**MTBF (hours)**	**Incidence (%)**
Ground Control System	2.00	500,000.0	6.62%
Mainframe	2.77	360,984.8	9.16%
Power plant	9.94	100,603.6	32.88%
Navigation system	9.41	106,269.9	31.13%
Electronic system	5.01	199,600.8	16.57%
Payload	1.10	909,090.9	3.64%
**λ TOTAL** =	**30.23**	**FIT**	
**MTBF (R_Total_)** =	**33,079.50**	**Hours**	
	**1378.31**	**Days**	
	**49.23**	**Months**	
**Military Drone (b)**			
**System Description**	**λ_P_ System FIT (F/10^6^ hrs)**	**MTBF (hours)**	**Incidence (%)**
Ground Control System	14.00	71,403.6	27.30%
Mainframe	2.77	360,984.8	5.40%
Power plant	21.08	47,428.7	41.10%
Navigation system	7.39	135,369.3	14.40%
Electronic system	3.44	290,942.9	6.70%
Payload	2.62	382,219.2	5.10%
**λ TOTAL** =	**51.30**	**FIT**	
**MTBF (R_Total_)** =	**19,493.18**	**Hours**	
	**812.22**	**Days**	
	**29.01**	**Months**	
